# Personal model‐assisted identification of NAD^+^ and glutathione metabolism as intervention target in NAFLD

**DOI:** 10.15252/msb.20167422

**Published:** 2017-03-02

**Authors:** Adil Mardinoglu, Elias Bjornson, Cheng Zhang, Martina Klevstig, Sanni Söderlund, Marcus Ståhlman, Martin Adiels, Antti Hakkarainen, Nina Lundbom, Murat Kilicarslan, Björn M Hallström, Jesper Lundbom, Bruno Vergès, Peter Hugh R Barrett, Gerald F Watts, Mireille J Serlie, Jens Nielsen, Mathias Uhlén, Ulf Smith, Hanns‐Ulrich Marschall, Marja‐Riitta Taskinen, Jan Boren

**Affiliations:** ^1^ Science for Life LaboratoryKTH – Royal Institute of TechnologyStockholmSweden; ^2^ Department of Biology and Biological EngineeringChalmers University of TechnologyGothenburgSweden; ^3^ Department of Molecular and Clinical MedicineUniversity of Gothenburg, and Sahlgrenska University HospitalGothenburgSweden; ^4^ Research programs UnitDiabetes and ObesityHelsinki University HospitalUniversity of HelsinkiHelsinkiFinland; ^5^ Department of RadiologyHUS Medical Imaging CenterHelsinki University Central HospitalUniversity of HelsinkiHelsinkiFinland; ^6^ Department of Endocrinology and MetabolismAcademic Medical CenterUniversity of AmsterdamAmsterdamThe Netherlands; ^7^ Department of Endocrinology–DiabetologyUniversity Hospital and INSERM CRI 866DijonFrance; ^8^ Faculty of EngineeringComputing and MathematicsUniversity of Western AustraliaPerthWAAustralia; ^9^ Metabolic Research CentreCardiovascular MedicineRoyal Perth HospitalSchool of Medicine and PharmacologyUniversity of Western AustraliaPerthWAAustralia

**Keywords:** glutathione, NAFLD, personalized genome‐scale metabolic modeling, serine, Genome-Scale & Integrative Biology, Metabolism, Systems Medicine

## Abstract

To elucidate the molecular mechanisms underlying non‐alcoholic fatty liver disease (NAFLD), we recruited 86 subjects with varying degrees of hepatic steatosis (HS). We obtained experimental data on lipoprotein fluxes and used these individual measurements as personalized constraints of a hepatocyte genome‐scale metabolic model to investigate metabolic differences in liver, taking into account its interactions with other tissues. Our systems level analysis predicted an altered demand for NAD ^+^ and glutathione (GSH) in subjects with high HS. Our analysis and metabolomic measurements showed that plasma levels of glycine, serine, and associated metabolites are negatively correlated with HS, suggesting that these GSH metabolism precursors might be limiting. Quantification of the hepatic expression levels of the associated enzymes further pointed to altered *de novo* GSH synthesis. To assess the effect of GSH and NAD^+^ repletion on the development of NAFLD, we added precursors for GSH and NAD ^+^ biosynthesis to the Western diet and demonstrated that supplementation prevents HS in mice. In a proof‐of‐concept human study, we found improved liver function and decreased HS after supplementation with serine (a precursor to glycine) and hereby propose a strategy for NAFLD treatment.

## Introduction

Hepatic steatosis (HS) is defined as the accumulation of fat in liver with no evidence of hepatocellular injury, and it is the most common chronic liver disease worldwide (Vetelainen *et al*, 2007). HS is the characteristic feature of non‐alcoholic fatty liver disease (NAFLD) and it is strongly associated with obesity, insulin resistance, type 2 diabetes (T2D), and cardiovascular diseases (Ratziu *et al*, 2010). Up to 30% of subjects with NAFLD develop non‐alcoholic steatohepatitis (NASH) which is a serious illness in which inflammation and scarring eventually can lead to cirrhosis and hepatocellular carcinoma (HCC) (Dyson *et al*, 2014).

The underlying molecular mechanisms leading to the occurrence of HS and its transition to severe liver disorders remain elusive, which limits the identification of drug targets and discovery of biomarkers that may be used to design effective treatment strategies. There are currently few pharmaceutical treatments for HS and its associated clinical conditions (Machado & Cortez‐Pinto, 2012), and an integrative systems biology‐based approach may help to address these significant unmet medical needs. In this context, genome‐scale metabolic models (GEMs) can be used to gain more insights about the molecular mechanisms involved in the occurrence of HS and associated disorders, and in turn may enable future therapeutic discoveries (Mardinoglu & Nielsen, 2012, 2015; Yizhak *et al*, 2013, 2014a,b; Zhang *et al*, 2015). GEMs are the collection of biochemical reactions that are known to occur in particular cells/tissues, and these models have been used in the integration of cellular, physiological, and clinical data to reveal the underlying molecular mechanisms of metabolism‐related disorders (Folger *et al*, 2011; Frezza *et al*, 2011; Mardinoglu *et al*, 2013a,b, 2014b, 2015a; Agren *et al*, 2014; Ghaffari *et al*, 2015; Varemo *et al*, 2015; Lee *et al*, 2016a,b). We have previously reconstructed a GEM for hepatocytes (*iHepatocytes2322*) and used it to analyze transcriptomics data obtained from NAFLD patients (Mardinoglu *et al*, 2014a). In addition, a GEM study showed that the liver adaptively regulates metabolic responses to maintain its basic functions and that NAFLD is associated with increased glyceroneogenesis and a switch from lactate to glycerol as a substrate for gluconeogenesis (Hyötyläinen *et al*, 2016).

To design effective treatment strategies for NAFLD, it is also necessary to understand the pathophysiology of dyslipidemia. Traditional approaches that measure plasma concentrations of lipoproteins provide only static estimates with limited mechanistic information. As an alternative, tracers labeled with stable isotopes can be used as a powerful tool for probing lipid and lipoprotein kinetics *in vivo* to provide an increased understanding about the pathogenesis of dyslipidemia (Boren *et al*, 2012; Adiels *et al*, 2015). The GEM *iHepatocytes2322* contains extensive information about lipid metabolism (Mardinoglu *et al*, 2014a), which is necessary for studying the effect of excess amount of lipids on the underlying molecular mechanism of NAFLD. This GEM can thus be used as a platform for studying the kinetics of lipoproteins and their potential effect on liver metabolism.

To clarify the underlying metabolic disturbances in NAFLD, we investigated the metabolic differences in liver between subjects with varying degrees of HS by studying the kinetics of lipid metabolism, taking into account interactions between the liver, adipose, muscle, and other peripheral tissues as well as red blood cells. Using personalized genome‐scale metabolic modeling, we elucidated an underlying molecular mechanism of NAFLD which can be used in the development of an effective treatment strategy.

## Results

### Characteristics of subjects with varying degrees of HS

**Figure 1:**
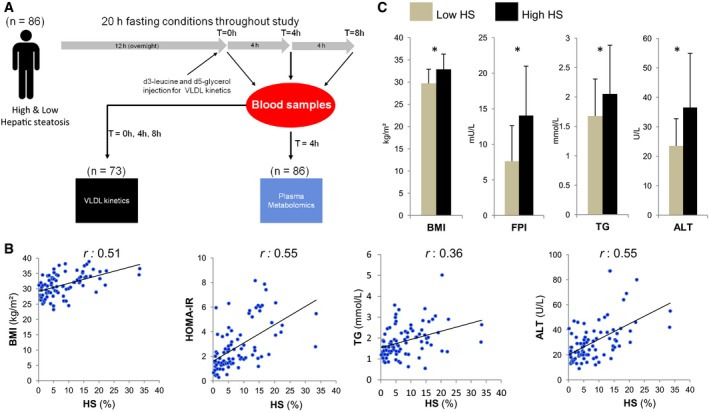
Generation of VLDL kinetics and plasma metabolomics data To identify the metabolic changes in response to increased hepatic steatosis (HS), secretion rate of the VLDLs from the liver of subjects was measured and the plasma metabolite levels were detected in subjects with varying degrees of HS.BMI, insulin resistance (HOMA‐IR), plasma TGs, and ALT levels are significantly correlated with the independently measured liver fat.The subjects were categorized into two groups as high (*n* = 43) and low HS (*n* = 43). Body mass index (BMI), fasting plasma insulin (FPI), plasma triglycerides (TGs), and plasma alanine aminotransferase (ALT) levels are found to be significantly different between the two groups. Data are presented as means ± SD. *P < 0.05; Student's *t*‐test.

We recruited 86 subjects (75 men and 11 women; Fig 1A) and determined the liver fat content of each subject using magnetic resonance spectroscopy (Adiels *et al*, 2006; Lundbom *et al*, 2011). The clinical characteristics of all the subjects involved in the study are presented in Table 1. We calculated the Pearson correlation coefficient (*r*) between HS and other clinical parameters and found that HS was significantly (*P* < 0.05) positively correlated with weight, body mass index (BMI), insulin resistance (HOMA‐IR), plasma triglyceride (TG), and the liver enzyme alanine aminotransferase (ALT) levels (Fig 1B). The ratio of ALT to aspartate transaminase (AST) also significantly (*P* < 0.05) correlated (*r* = 0.57) with HS. None of the other liver‐related clinical parameters (AST, alkaline phosphatase (ALP), and γ‐glutamyl transferase (GT)), blood lipid‐related parameters (high‐density lipoprotein (HDL) cholesterol, total cholesterol, and apolipoprotein B (apoB)) nor the inflammation marker C‐reactive protein (CRP) correlated significantly with HS.

**Table 1:** Clinical characteristics of the 86 study participants

Characteristic	Low HS HS (%) < 5.5 *n* = 43	High HS HS (%) > 5.5 *n* = 43	*P*‐value
Liver fat (%)	2.8 ± 1.7	13.4 ± 6.4	**<0.05**
Age (years)	52.1 ± 8.4	52.6 ± 8.0	0.76
Weight (kg)	92.4 ± 11.2	102.2 ± 14.0	**<0.05**
Body mass index (BMI) (kg/m²)	29.7 ± 3.2	32.9 ± 3.4	**<0.05**
Fasting plasma glucose (mmol/l)	5.5 ± 0.5	5.85 ± 0.6	**<0.05**
Fasting plasma insulin (FPI) (mU/l)	7.6 ± 5.0	14.0 ± 7.0	**<0.05**
HOMA‐IR	1.9 ± 1.3	3.7 ± 2.0	**<0.05**
C‐reactive protein (CRP) (mg/l)	2.4 ± 2.8	3.3 ± 3.3	0.31
Plasma triglycerides (TG) (mmol/l)	1.7 ± 0.6	2.1 ± 0.8	**<0.05**
Apolipoprotein B (apoB) (mg/dl)	90.2 ± 29.0	97.4 ± 30.7	0.30
Total cholesterol (mmol/l)	4.9 ± 0.85	5.1 ± 0.7	0.31
HDL cholesterol (mmol/l)	1.1 ± 0.3	1.1 ± 0.3	0.96
Alanine aminotransferase (ALT) (U/l)	23.5 ± 9.3	38.9 ± 28.3	**<0.05**
Aspartate aminotransferase (AST) (U/l)	21.9 ± 5.1	23.2 ± 5.8	0.41
Alkaline phosphatase (ALP) (U/l)	63.2 ± 16.7	70.5 ± 19.3	0.16
γ‐glutamyl transferase (GT) (U/l)	27.6 ± 16.4	31.2 ± 13.9	0.40

Data are presented as means ± SD. *P*‐value indicates significance level of difference between the subjects with low and high hepatic steatosis (HS). *P*‐values were calculated using Student's *t*‐test. Bold text indicate significantly different values.

We classified the subjects with varying degrees of HS into two groups of 43 subjects based on their liver fat percentage: high HS (> 5.5%) and low HS (< 5.5%) (Table 1). We found that subjects with high HS were significantly (*P* < 0.05) heavier with a greater BMI. Fasting plasma glucose and fasting plasma insulin (FPI) concentrations were significantly (*P* < 0.05) higher in subjects with high HS compared to subjects with low HS (Fig 1C). The average plasma TG concentration was 2.05 and 1.67 mmol/l for subjects with high and low HS, respectively (Fig 1C). We did not detect any significant plasma differences in other lipid parameters including apoB, HDL cholesterol, and total cholesterol (Table 1). The ALT level was significantly higher in subjects with high HS (Fig 1C). In summary, the average subject with low HS involved in our study was overweight, borderline hypertriglyceridemic but insulin sensitive, whereas the average subject with high HS was obese, hypertriglyceridemic and insulin resistant but did not have T2D.

### Personalized liver tissue GEMs

**Figure 2:**
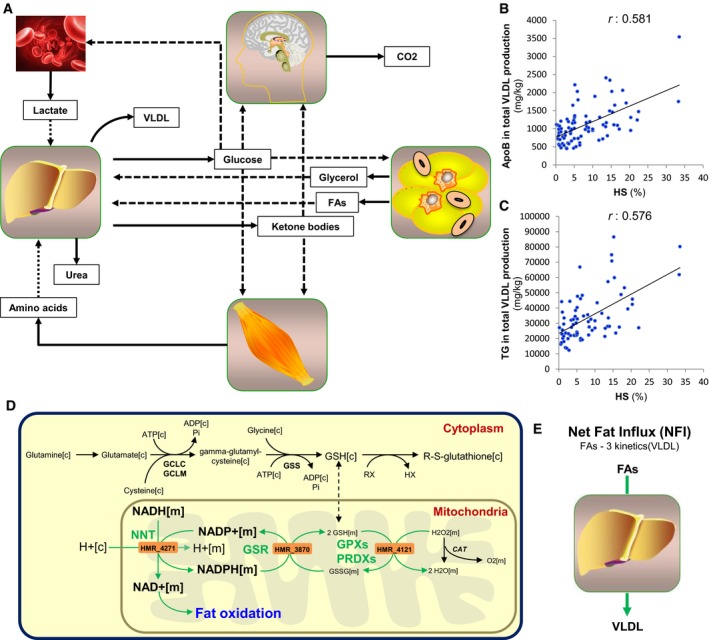
Personalized modeling of liver in subjects with varying degrees of HS ASchematic illustration of how personalized genome‐scale metabolic modeling can be performed accounting the interactions between other tissues and red blood cells for the development of effective therapeutic approaches for non‐alcoholic fatty liver disease (NAFLD). Solid and dashed arrows show the outputs and inputs to the tissues, respectively.B, CThe correlation between the predicted intracellular fluxes of the liver and hepatic steatosis (HS) is assessed and compared with the (B) apolipoprotein B (apoB) and (C) triglycerides (TG) content in the total VLDL production.DThe fluxes carried by the reactions catalyzed by NNT, GSR, and GPXs are found to be the most correlated reactions with the HS (Dataset EV3). Green arrow indicates the significant correlation of the flux carried by the reactions and HS.EThe net fat influx (NFI) is calculated as the differences in the uptake and secretion rates of FAs and its correlation with the intracellular fluxes are assessed.

To elucidate the underlying molecular mechanisms of HS, we adopted constraint‐based modeling techniques to identify major hepatic metabolic alterations between subjects with varying degrees of HS. The secretion rates of non‐esterified fatty acids (FAs) and amino acids (AAs) from adipose and muscle tissues were calculated based on the body composition of each subject and used as input to the personalized liver GEMs together with the lactate secreted by red blood cells (Fig 2A and Materials and Methods, and Dataset EV1). Since the level of TG‐rich very‐low‐density lipoproteins (VLDLs) is the major determinant of plasma TG, we combined kinetic studies with stable isotopes and multicompartment modeling to infer the parameters of VLDL kinetics in 73 of the subjects (65 men and 8 women) involved in our study. We observed a significant correlation between secreted VLDL and HS (*r *= 0.581, *P* < 0.001) and used secretion rate of VLDL as an objective function for the personalized liver GEMs (Dataset EV1).

We simulated the dynamics of the liver metabolism that would ideally correspond to the increased HS using the inputs and outputs to the liver as constraints of personalized GEMs. We predicted the intracellular fluxes in the liver of each subject (Dataset EV2) and calculated the Pearson correlation coefficient between the intracellular fluxes and HS of each subject (Dataset EV3). We found that reactions involved in protein synthesis had the highest correlations with HS (*r* = 0.57, *P* < 0.001). We also quantified the apoB content in the total VLDL produced by the liver and found that it correlated significantly with the measured HS (*r *= 0.581, *P* < 0.001) (Fig 2B). This correlation was very similar to that observed between the TG content in the total VLDL produced and the measured HS (*r *= 0.576, *P* < 0.001) (Fig 2C). Hence, we observed that personalized GEMs were able to predict the response of liver to the increased HS.

Reactions with the second and third highest correlations with HS were those involved in the reduction of H_2_O_2_ (*r *= 0.482, *P* < 0.001) and those associated with nicotinamide nucleotide transhydrogenase (NNT) (*r *= 0.479, *P* < 0.001), respectively. NNT catalyzes the interconversion of NADH and NADP^+^ to NAD^+^ and NADPH in the mitochondria. NNT has an important role in providing NAD^+^ for fat oxidation as well as NADPH for redox detoxification since NADPH is used for the regeneration of glutathione (GSH) through reduction of glutathione disulfide (GSSG), which is catalyzed by glutathione reductase (GSR) (Fig 2D). Notably, we found that the flux carried by the reaction associated with GSR was one of those with the highest correlations with HS (*r *= 0.478, *P* < 0.001). Moreover, we found that reactions involved in fat oxidation significantly correlated with HS (*r *= 0.477, *P* < 0.001) (Dataset EV3). Increases in the flux carried by the reactions catalyzed by NNT and GSR would generate additional NAD^+^, which is necessary for the increased fat oxidation and GSH, which is necessary to scavenge excessively produced reactive oxygen species resulting from increased fat oxidation. It has previously been reported that NNT is essential for normal cellular metabolism and for mitochondrial defense against oxidative stress (Huang *et al*, 2006). In addition, we observed a significant correlation between HS and secreted ketone bodies, which are one of the major outputs of the liver GEM (*r *= 0.475, *P* < 0.001) (Dataset EV3).

Hepatic steatosis results from an imbalance between the *de novo* synthesis, oxidation, uptake, and export of FAs (Tamura & Shimomura, 2005). Hence, we calculated the differences in the uptake and secretion rates of FAs, defined as net fat influx (NFI) (Fig 2E), in the liver of each subject, and calculated the correlations between the intracellular fluxes and NFI (Dataset EV4). Notably, we found that the reactions catalyzed by GSR (*r *= 0.812, *P* < 0.001) and NNT (*r *= 0.811, *P* < 0.001) had the highest correlations with NFI. We also found that the reaction catalyzed by glutathione peroxidases (GPXs) and peroxiredoxins (PRDXs) (Fig 2D), which detoxify peroxides and hydroperoxides, was significantly correlated with NFI (*r *= 0.812, *P* < 0.001) (Dataset EV4). In addition, we observed a significant correlation between NFI and secreted ketone bodies (*r *= 0.782, *P* < 0.001).

Increased GSH and NAD^+^ formation as well as the increased fat oxidation in our *in silico* analysis are model‐predicted demands which would ideally be met for dealing with high HS. If these demands cannot easily be met *in vivo* due to reduced concentrations of the substrates, then cellular health might be compromised. Considering that our simulations demonstrated the ideal response of the liver to the increased HS, the upregulation of the fat oxidation as well as the increased availability of the GSH and NAD^+^ may provide potential treatment strategy for NAFLD subjects.

### Glycine is the limiting substrate for *de novo* synthesis of GSH in NAFLD

Depletion of GSH can lead to mitochondrial dysfunction and cell death (Fernandez‐Checa & Kaplowitz, 2005; Garcia‐Canaveras *et al*, 2011). Based on our *in silico* analysis, we proposed that an increased expression of NNT would be needed to boost the level of NAD^+^ to respond to the increased fat oxidation whereas NNT and GSR may boost the level of GSH required for resisting oxidative stress and maintaining the reducing environment of the liver. However, if the expression of NNT and GSR cannot meet this demand or even decrease in the pathological state, it may result in depletion of NAD^+^ and GSH and eventually to accumulation of fat in the liver. Indeed, hepatic depletion of NAD^+^ in mice model of NAFLD has been reported (Gariani *et al*, 2016; Zhou *et al*, 2016). Moreover, lower concentrations of both GSH and GSSG and a reduction in the GSH/GSSG ratio have been reported in the liver (Garcia‐Canaveras *et al*, 2011) and serum (Kalhan *et al*, 2011) of NAFLD patients compared to healthy subjects.

**Figure 3:**
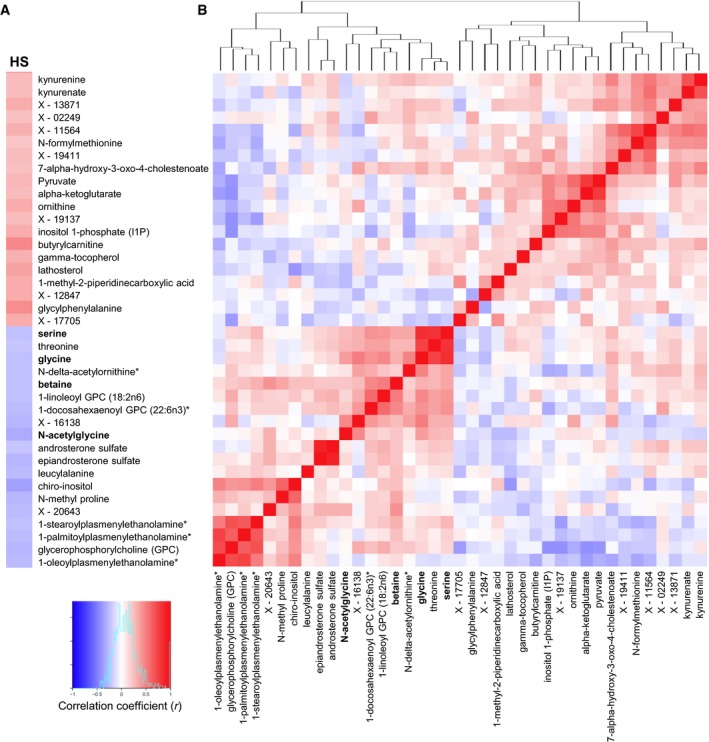
Correlation of the HS and plasma metabolomics data A, BHepatic steatosis (HS) is measured by the magnetic resonance imaging, and the plasma level of ˜520 metabolites was detected by untargeted metabolomics profiling. (A) The correlation between the HS and the plasma metabolites and (B) the Pearson correlation between the significantly (*P* < 0.05) correlated metabolites are presented. Red and blue colors represent the positive and negative correlation of the HS and plasma metabolite levels, respectively. Metabolites that can be converted to glycine marked bold.

Depleted GSH can also be replaced by *de novo* synthesis of GSH from glutamine, glycine, and cysteine which can be taken up from the plasma. To detect the plasma level of these AAs, we performed non‐targeted metabolomics profiling in plasma from 86 subjects and analyzed levels of ~520 metabolites. We assessed the correlations between the plasma metabolite levels and HS (Dataset EV5). Fasting plasma levels of glycine and *N*‐acetylglycine as well as betaine and serine (which can be converted to glycine) showed significantly negative correlations with HS (Fig 3A). We also assessed the correlation coefficients between the plasma metabolites that correlated significantly with HS (Fig 3B) and found that plasma glycine levels showed the highest correlation with the plasma serine levels among all other measured metabolites (*r *= 0.77, *P* < 0.05) (Dataset EV5). It should be noted that we did not detect any significant correlation between HS and the plasma levels of cysteine and glutamine (which are also required for the *de novo* synthesis of GSH).

**Figure 4:**
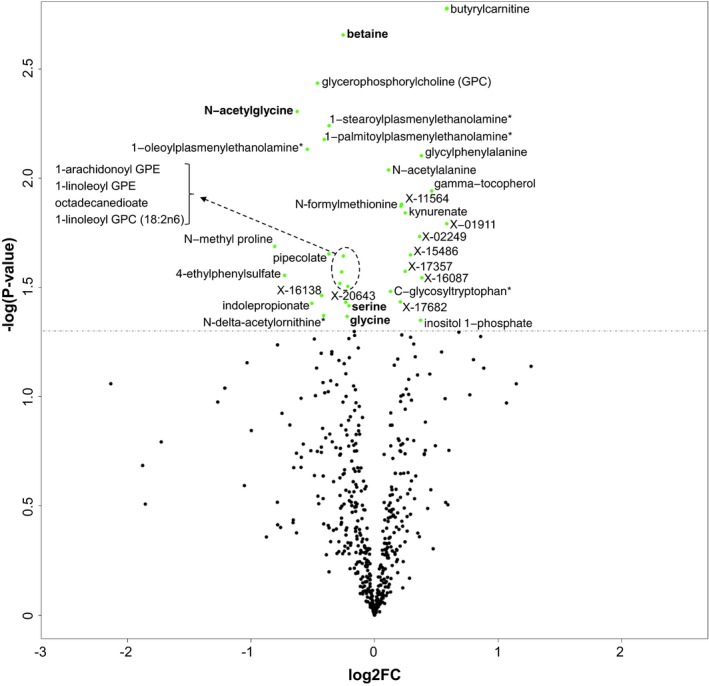
Identification of significantly changed metabolites in subjects with high HS The plasma level of ~520 metabolites was detected by untargeted metabolomics profiling and significantly (*P* < 0.05) changed metabolites are presented using volcano plot. Metabolites that can be converted to glycine marked bold.

We also investigated whether any of the plasma metabolites showed significant differences between the two groups of subjects divided according to their level of HS (Dataset EV6). We found that the levels of glycine, serine, betaine, and *N*‐acetylglycine were significantly (Welsh's *t*‐test, *P* < 0.05) lower in subjects with high HS compared to those with low HS (Fig 4). In addition to the metabolites associated with glycine, we also found that the levels of butyrylcarnitine, glycylphenylalanine, gamma‐tocopherol (vitamin E), kynurenate, N‐delta‐acetylornithine, *N*‐methyl proline, and a number of lipid structures, which have been shown to correlate with HS in Fig 3 were significantly changed (Welsh's *t*‐test, *P* < 0.05) between the subjects with high and low HS (Fig 4). Changes in the plasma levels of butyrylcarnitine, glycine as well as some of other metabolites identified in our study have previously been observed in studies that compared NAFLD subjects to healthy subjects (Kalhan *et al*, 2011; Dumas *et al*, 2014).

### Decreased expression of the enzymes involved in GSH formation

**Figure 5:**
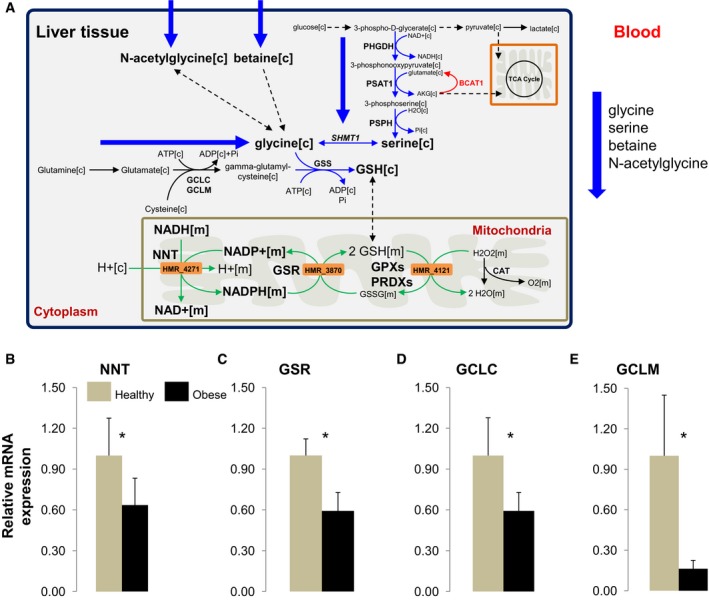
Glycine is the limiting substrate in the synthesis of GSH in NAFLD AGlycine is found to be the limiting substrate for the synthesis of glutathione (GSH) in subjects with NAFLD. Decreased *de novo* synthesis of serine has also been reported in subjects with NASH. The decreased plasma level of glycine, serine as well as other associated metabolites betaine and *N*‐acetylglycine in subjects with high HS is confirmed with the metabolomics study. In order to confirm model‐based predictions, serine was supplemented to the subjects with high HS since serine‐derived carbon can be converted to GSH to satisfy the increased demand for GSH in NAFLD. Blue arrows indicated downregulation whereas the red arrow indicates upregulation of the reactions. Green arrows indicate the significant correlation of the flux carried by the reactions and HS. [c], cytoplasm; [m], mitochondria.B–EThe mRNA expressions of the (B) nicotinamide nucleotide transhydrogenase (NNT), (C) glutathione reductase (GSR), (D) glutamate–cysteine ligase, catalytic subunit (GCLC), and (E) glutamate–cysteine ligase, modifier subunit (GCLM) are measured in the livers obtained from 12 morbidly obese subjects who had undergone bariatric surgery and seven healthy individuals (mean ± SD). *P < 0.05; Student's *t*‐test.

We revealed that a disturbed redox balance (i.e., NAD^+^ and GSH deficiency) was linked to metabolic dysfunction and development of NAFLD. In this context, we measured the expression of NNT, GSR, and the enzymes involved in the *de novo* GSH synthesis in human liver samples obtained from a separate cohort of 12 obese subjects with high HS who underwent bariatric surgery (Table 2) and compared with the expression of the genes in liver samples obtained from seven healthy individuals (previously described in Uhlen *et al*, 2015) (Fig 5A). We found that mRNA expression of NNT, GSR, and the rate‐limiting enzymes in *de novo* GSH synthesis, namely glutamate–cysteine ligase, catalytic subunit (GCLC) and glutamate–cysteine ligase, modifier subunit (GCLM), were significantly lower in liver from obese subjects than from healthy subjects (Fig 5B–E). This indicated that the decreased expression of the NNT and GSR may lead to increased HS which is in agreement with the results of personalized modeling of subjects with varying degree of HS.

**Table 2:** Clinical characteristics of the twelve obese subjects who underwent bariatric surgery with high HS

Clinical variable	Obese subjects with high HS (*n* = 12)
Age (years)	39.3 ± 10.9
Weight (kg)	122.9 ± 12.8
Body mass index (BMI) (kg/m²)	43.6 ± 3.6
Fasting plasma glucose (mmol/l)	5.6 ± 0.6
Fasting plasma insulin (FPI) (pmol/l)	128.7 ± 49.9
HOMA‐IR	4.7 ± 1.9
Plasma triglycerides (TG) (mmol/l)	1.5 ± 0.5
Total cholesterol (mmol/l)	5.1 ± 0.7
LDL cholesterol (mmol/l)	3.1 ± 0.7
HDL cholesterol (mmol/l)	1.3 ± 0.3
Alanine aminotransferase (ALT) (U/l)	25.3 ± 16.3
γ‐glutamyl transferase (GT) (U/l)	30.7 ± 23.2

Data are presented as means ± SD.

### Supplementation of GSH and NAD^+^ precursors decreases HS in mice

Our analysis indicated depletion of the NAD^+^ and GSH in subjects with high HS. It has been shown that supplementation of natural NAD^+^ precursors, such as tryptophan, nicotinamide riboside (NR), niacin, and nicotinamide, elevates NAD^+^ levels *in vivo* (Houtkooper *et al*, 2010; Canto *et al*, 2012). The plasma and liver level of GSH is depleted in NAFLD patients and cannot be increased by supplementation with GSH; instead, GSH must be synthesized within the liver either *de novo* or by the salvation pathway. Our analysis suggested that the level of GSH is not sufficient to maintain and regulate the thiol‐redox status of the liver in subjects with high HS in the fasting state due to the depletion of glycine. Glycine can be synthesized via the interconversion of serine through serine hydroxymethyltransferases with concomitant conversion of tetrahydrofolate (THF) into 5,10‐methylene‐THF (Fig 5A). During the conversion of serine to glycine, an additional carbon unit is provided for one‐carbon metabolism. Previously, we have shown that serine synthesis is downregulated in NASH patients (Mardinoglu *et al*, 2014a). Taken together, we therefore hypothesized that dietary supplementation with NR may increase the level of NAD^+^ required for the increased fat oxidation and serine may increase the level of glycine and the level of GSH (by intracellular GSH synthesis from glycine). Supplementation of the substrates for NAD^+^ and GSH may increase the amount of the fat oxidized in the liver, lower oxidative stress resulting from high fat oxidation, lower the level of HS, and eventually improve liver function.

**Figure 6:**
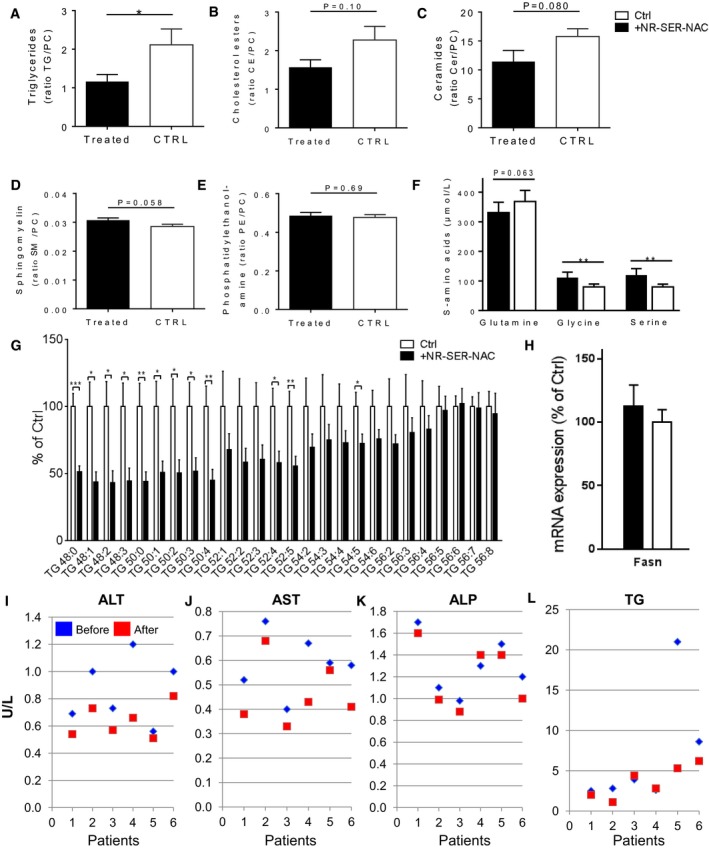
Supplementation of NAD^+^ and GSH precursors prevent NAFLD Ten mice were given the Western diet supplemented with NR (400 mg/kg/day) and serine (300 mg/kg/day) *la gavage* and NAC (1 g/l) in the drinking water, and ten mice were only given the Western diet for 14 days. 
A–EHepatic lipids including (A) triglycerides (TG), (B) cholesterol esters (CE), (C) ceramides (CER), (D) sphingomyelin (SM), (E) phosphatidylethanolamine (PE) (normalized to phosphatidylcholine (PC)) are shown in treated (cocktail supplemented) (*n* = 10), and control (*n* = 10) mice (mean ± SEM).FQuantification of amino acids from the liver of the same mice before and after supplementation (mean ± SEM).GAnalysis of the molecular species of TGs extracted from the livers of the mice. Results from the control group are expressed as 100%, and results from the treated group are expressed as % of the control group (mean ± SEM).HmRNA expression of the fatty acid synthase (Fasn) in the liver tissue of mice before and after the supplementation. Six subjects received one oral dose of L‐serine (200 mg/kg/day) for 14 days (mean ± SEM).I–LThe human plasma (I) alanine aminotransferase (ALT), (J) aspartate aminotransferase (AST), (K) alkaline phosphatase (ALP), and (L) TGs levels are presented in each human subject involved in the study before and after the supplementation with serine.Data information: (A–G) *P < 0.05, **P < 0.01, ***P <0.001; Student's *t*‐test.Source data are available online for this figure.

To assess the effect of GSH and NAD^+^ repletion on the development of HS in mice, we supplemented a cocktail consisting of serine, NAC (*N*‐acetyl‐L‐cysteine), and NR to mice fed with Western diet, including high levels of fat and sucrose. Serine was included into the cocktail since it can be easily converted to glycine whereas NAC was included since cysteine may be the limiting metabolite after the repletion of the glycine in the synthesis of GSH. NR was included in the cocktail to increase the amount of NAD^+^ in the liver. We treated Western diet‐fed male C57BL/6J mice with serine (300 mg/kg/day) and NR (400 mg/kg/day) *la gavage* as well as NAC (1g/l) in the drinking water for 14 days and sacrificed the mice 4 h after the last treatment. We obtained liver tissue samples from the mice, performed liver lipidomics analysis and observed 50% reduction in hepatic TGs (Fig 6A), tendency to decrease in the level of cholesterol esters (Fig 6B) and ceramides (Fig 6C), and tendency to increase in the level sphingomyelin (Fig 6D) and no significant changes in the level of phosphatidylethanolamine (Fig 6E). We also measured the level of glycine and serine and found that their plasma level was significantly increased after supplementation of the cocktail (Fig 6F). We finally measured the liver level of TGs with different chain lengths and found that shorter chain lengths of TGs that are preferentially oxidized in the mitochondria were significantly decreased after supplementation (Fig 6G). Hence, we demonstrated that supplementation of the metabolites that are predicted by personalized modeling promotes the oxidation of fat in the liver, prevents HS, and hereby proposed a therapeutic strategy for protecting against NAFLD progression.

Fat oxidation and fatty acid synthesis are reciprocal metabolic pathways (Foster, 2012). In order to check whether the *de novo* lipogenesis is inhibited by the supplementation of the cocktail, we measured the expression of the fatty acid synthase (Fasn), a key enzyme involved in the *de novo* lipogenesis in the liver tissue of the mice but did not find any significant differences in its expression before and after the supplementation of the cocktail (Fig 6H).

### Supplementation of serine decreases HS in humans

To identify the unique contribution of serine supplementation in decreasing HS, we assessed the effect of short‐term dietary supplementation with serine on HS and fasting levels of plasma markers of liver functions in six subjects with high HS. Characteristics of the six subjects before and after the supplementation are presented in Table 3. Each patient received one oral dose of ~20 g of L‐serine (200 mg/kg/day) for 14 days. The supplementation was well tolerated by all the subjects. We found that plasma ALT, AST, and ALP levels were significantly decreased after supplementation (Table 3). Notably, we found that the plasma levels of ALT (Fig 6I) and AST (Fig 6J) were consistently decreased in all six subjects and ALP was decreased in five of the participating subjects (Fig 6K). Moreover, we found that the plasma TGs were decreased in five of the studied subjects and did not change in the remaining one subject (Fig 6L). We finally measured the HS using magnetic resonance spectroscopy before and after serine supplementation and demonstrated that HS is significantly decreased after serine supplementation (Table 3). HS is decreased in all six patients, and the relative decrease in NAFLD patients ranged between 1.0 and 23%.

**Table 3:** Clinical characteristics of the six subjects involved in serine supplementation study

Clinical variable	Baseline (*n* = 6)	After serine (*n* = 6)	*P*‐value
Liver fat (%)	26.8 ± 6.0	20.4 ± 7.0	**<0.05**
Age (years)	56.7 ± 5.2	56.7 ± 5.2	**–**
Weight (kg)	103.0 ± 14.3	103.0 ± 13.9	**–**
Body mass index (BMI) (kg/m²)	32.5 ± 2.70	32.5 ± 2.60	**–**
Alanine aminotransferase (ALT) (U/l)	50.8 ± 15.2	37.6 ± 5.3	**<0.05**
Aspartate aminotransferase (AST) (U/l)	34.5 ± 8.10	27.4 ± 8.4	**<0.05**
Alkaline phosphatase (ALP) (U/l)	76.3 ± 17.2	71.3 ± 17.9	**<0.05**
γ‐glutamyl transferase (GT) (U/l)	63.8 ± 12.9	62.3 ± 16.3	0.30
Fasting plasma glucose (mmol/l)	6.57 ± 1.41	6.33 ± 1.41	0.25
Fasting plasma insulin (FPI) (pmol/l)	46.3 ± 33.8	34.7 ± 25.2	0.23
HOMA‐IR	2.15 ± 1.85	1.54 ± 1.49	0.18
LDL cholesterol (mmol/l)	3.68 ± 0.80	3.85 ± 0.94	0.50
HDL cholesterol (mmol/l)	1.00 ± 0.21	1.02 ± 0.18	0.30
Plasma triglycerides (TG) (mmol/l)	6.90 ± 6.65	3.63 ± 1.81	0.13
Total cholesterol (mmol/l)	6.23 ± 1.49	5.85 ± 1.15	0.18
Bilirubin (μmol/l)	7.33 ± 4.11	6.48 ± 3.94	0.13

Data are presented as means ± SD. *P*‐value (calculated using Student's *t*‐test) indicates the significance level of difference before and after the oral supplementation of serine. Bold text indicate significantly different values.

## Discussion

Personalized genome‐scale metabolic modeling provides deeper insight into clinical data, enabling increased understanding of the genotype–phenotype relationship. We characterized subjects with varying degrees of HS and measured VLDL kinetics. Subsequently, we integrated the VLDL kinetic data with additional experimentally derived flux data to simulate the desired dynamics of liver metabolism of each subject using a liver GEM. We then assessed the correlations between the predicted intracellular fluxes of the liver and HS to investigate whether any metabolic derangements could be detected in NAFLD. Our systems level analysis indicated that altered NAD^+^ and GSH metabolism (with increased demand for NAD^+^ and GSH in NAFLD) was a prevailing feature in NAFLD. Hence, we postulated that subjects with NAFLD have reduced *de novo* synthesis of GSH, possibly due to limited availability of glycine in the fasting state. We analyzed plasma metabolomics and showed that plasma levels of glycine as well as serine, betaine, and *N*‐acetylglycine (which can be converted to glycine) were lower in subjects with high HS compared to those with low HS. Moreover, analysis of the metabolomics data revealed significant negative correlations between the plasma levels of glycine, serine, betaine, and *N*‐acetylglycine with HS. In a mice study, we showed that supplementation of the precursors for NAD^+^ and GSH significantly decreased HS. Finally, in a proof‐of‐concept human study, we found that HS is significantly decreased whereas markers of liver function are significantly improved in NAFLD patients after supplementation with serine (a precursor to glycine).

Our finding highlighting the importance of glycine is consistent with an earlier pilot study which showed that supplementation with betaine (which is degraded to sarcosine and glycine) resulted in significant biochemical and histological improvements in patients with NASH (Abdelmalek *et al*, 2001). Furthermore, mice deficient in glycine *N*‐methyltransferase (Gnmt), which is involved in the degradation of betaine to glycine, have hyperlipidemia and steatohepatitis (Liao *et al*, 2012). In addition, glycine supplementation to rats with alcohol‐induced liver injury has been shown to protect against free radical‐mediated oxidative stress in hepatocytes (Senthilkumar *et al*, 2004).

Serine derived from a branch of glycolysis can be converted to glycine, which in turn provides carbon units for one‐carbon metabolism using THF. It has been shown that NAFLD patients and controls have similar folate levels (de Carvalho *et al*, 2013), and THF is therefore not likely to be limiting for glycine biosynthesis. In this context, serine supplementation to mice and rats has been shown to attenuate alcoholic fatty liver by enhancing homocysteine metabolism (Sim *et al*, 2015).

Increased release of free FAs in the fasting state is a known characteristic of obesity and associated disorders such as NAFLD (Nestel & Whyte, 1968; Karpe *et al*, 2011). We showed that the influx of FAs into the liver with simultaneous low secretion of VLDL (i.e., high NFI) profoundly affected the fluxes. GSH turnover as well as increased fat oxidation, increased oxidative phosphorylation with subsequent increased demand for oxygen, and increased ketogenesis were strongly correlated with high NFI. However, it should be noted that although the flux of the reactions involved in these pathways were able to increase in response to increased demand according to our *in silico* model, this might not be the case *in vivo*. Hence, the increases in GSH, NAD^+^, fat oxidation, oxygen consumption, and ketone production are thus all model‐predicted demands which would ideally be met for dealing with high HS. For example, if the predicted demand for GSH in high HS is not met by an increased supply of GSH, then the redox balance could be at risk of being insufficient for normal cellular health in high HS. Indeed, we showed the mRNA expression of the enzymes involved in the formation of GSH is significantly lower in obese subjects.

The subjects at highest risk of possible metabolic stress in this analysis were subjects with high FA influx and HS. Importantly, HS alone was not the single characteristic that explained higher demand for GSH, meaning a person with high HS is not necessarily at risk. Since metabolic distress was predicted to correlate well with high NFI and FA influx alone, it can therefore be argued that a subject with high HS but low FA influx is not necessarily at risk of disease. In fact, the expansion of intracellular stored lipids in the liver is one way of disposing of excess FAs. Thus, the HS process itself could theoretically serve to decrease metabolic stress in the liver. Similarly, increased VLDL secretion, increased ketone secretion, and increased oxidative phosphorylation are all means through which the liver can dispose of excess FAs.

Through systems level analysis in mice, we also observed that glycine is the limiting substrate for the *de novo* synthesis of GSH (Mardinoglu *et al*, 2015b). In a recent study comparing germ‐free and conventionally raised mice, we showed that the gut microbiota alters the distribution of AAs along the gastrointestinal tract, affecting the bioavailability of free AAs to the host (Mardinoglu *et al*, 2015b). We also showed that microbiota‐induced imbalances in the utilization of AAs, particularly serine and glycine, may affect the biological function of the host. Moreover, the presence of a gut microbiota resulted in increased expression of Nnt in the liver, adipose, and gastrointestinal tract tissues and a parallel decrease in plasma and liver levels of glycine.

There is increasing interest in identifying patients at high risk of developing NASH and, if possible, offering early treatment to prevent the development of the disease. Our data indicate that increased FA release from adipose tissue and decreased VLDL secretion from the liver elevate the metabolic stress on the liver. Therefore, it would potentially be of clinical value to take into account FA release from adipose tissue together with the degree of HS in subjects with HS.

**Figure 7:**
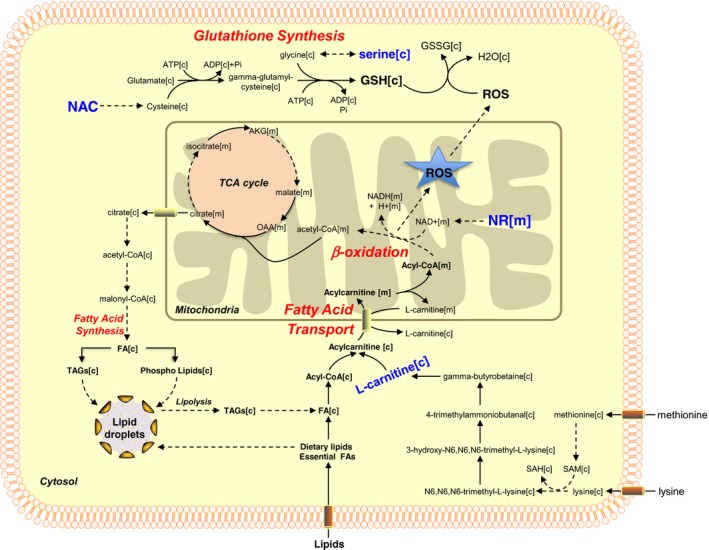
Three‐step strategy for the treatment of NAFLD and associated disorders Based on our results, we postulate a potential treatment strategy for NAFLD patients based on increased oxidation of fat and increased synthesis of GSH. A cocktail can be supplemented to NAFLD patients to boost these metabolic processes and to decrease the hepatic lipid accumulation. NR can be supplemented to boost the oxidation of the fat in the mitochondria by generating NAD^+^. Serine and NAC can be included in the cocktail to boost the level of GSH which is required for preventing the accumulation of incomplete products of fatty acids oxidation. l‐carnitine can also be added to the cocktail to boost the fatty acid uptake into mitochondria.

Through personalized modeling of the subjects with varying degree of HS, we observed that liver has capacity to clear the accumulated fatty acids by oxidizing them in the liver. Based on our analysis, a three‐step strategy including (i) increasing fatty acid uptake into mitochondria, (ii) increasing the oxidation of the fatty acids in the mitochondria, and (iii) increasing the availability of GSH can be employed to treat the subjects with high HS (Fig 7). A cocktail can be supplemented to boost these metabolic processes to decrease the amount of hepatic lipids. l‐carnitine and NR would stimulate the transfer of fatty acids from cytosol to mitochondria and boost the level of NAD^+^ which is required for mitochondrial fatty acid oxidation. Impaired function of the electron transport chain combined with increased rates of fatty acid oxidation may lead to the accumulation of incomplete products of fatty acid oxidation, which combined with increased levels of reactive oxygen species, may contribute to insulin resistance (Loh *et al*, 2009). To avoid this, the level of GSH can be increased by including serine and NAC into the content of the cocktail. This three‐step strategy may be useful to increase the level of fatty acids oxidized in the liver, deal with the excess amount of oxygen radicals resulted from increased fat oxidation, protect against free radical‐mediated oxidative stress, and eventually decrease HS in NAFLD patients. Such cocktail can also be used for the treatment of alcoholic fatty liver disease (AFLD) patients since HS is the earliest abnormality in the pathogenesis of both AFLD and NAFLD due to metabolic risk factors associated with insulin resistance and/or metabolic syndrome in the presence or absence of alcohol consumption (Lakshman *et al*, 2015). Considering that NAFLD and T2D are common conditions that regularly coexist and can act synergistically to drive adverse outcomes (Hazlehurst *et al*, 2016), such cocktail can also be used in the treatment of the subjects with T2D.

In conclusion, we used personalized genome‐scale metabolic modeling to elucidate molecular mechanisms involved in the progression of NAFLD and validated our predictions by generating additional transcriptomics and metabolomics data. In addition, we performed proof‐of‐concept studies in mice and human and showed that supplementation of the precursors for NAD^+^ and GSH may be useful in preventing and treatment of HS. Thus, personalized GEMs provide a useful new tool for elucidating the underlying mechanisms of disturbed liver metabolism in subjects with high HS and proposed a strategy for treatment of NAFLD.

## Materials and Methods

### Subjects

We recruited 86 subjects with varying degrees of HS for studying the response of liver to the HS. The clinical characteristics of the subjects are presented in Table 1. We also collected liver tissue samples from 12 morbidly obese subjects underwent bariatric surgery and presented the subjects characteristics in Table 2. We measured the mRNA expression of the identified target genes in liver of obese and healthy subjects.

In order to show the effect of the serine on the treatment of NAFLD, we recruited another six subjects and presented the subjects characteristics in Table 3 before and after serine supplementation. Each patient received one oral dose of ~20 g of l‐serine (200 mg/kg/day) for 14 days. Subjects included in our studies met all the criteria for NAFLD including exclusion of other chronic liver diseases such as viral hepatitis, risky alcohol consumption, and metabolic disorders (e.g., hemochromatosis). The supplementation study has been submitted to https://ClinicalTrials.gov with the identifier: NCT02599038.

### Determination of liver, subcutaneous, and intra‐abdominal fat

Magnetic resonance experiments were performed using three 1.5 T clinical imagers (1× Sonata and 2× Avanto, Siemens, Erlangen, Germany). Liver fat content was determined using proton magnetic resonance spectroscopy, and subcutaneous abdominal and visceral fat was measured by magnetic resonance imaging (Adiels *et al*, 2006; Lundbom *et al*, 2011).

### Measurement of flux data

Lipoprotein fluxes were measured in fasted 73 subjects using stable isotope infusion. After a bolus infusion of d3‐leucine and d5‐glycerol, large (VLDL1) and small (VLDL2) VLDL subfractions were isolated by ultracentrifugation and the enrichment of free leucine in plasma, leucine in apoB, and glycerol in TG was measured using gas chromatography–mass spectrometry (Adiels *et al*, 2005). Metabolic fluxes were calculated using mathematical modeling as previously described (Adiels *et al*, 2005).

### Muscle mass and fat mass calculations

The muscle mass of each subject was calculated from lean mass using the previously described relationship (Clark *et al*, 2014) based on their fat mass. A linear equation was fitted between BMI and fat mass of the 44 subjects to predict the missing fat mass in the remaining 29 subjects. The linear equation was defined as: fat mass (kg) = 1.763 × BMI − 26.75 (*R* ^2^ = 0.69). Using this equation, fat mass was calculated for the 29 subjects and the lean mass was then calculated by subtracting the fat mass from the body weight of the subjects. Finally, the muscle mass for each subject was calculated based on the previously derived equation (Clark *et al*, 2014): muscle mass = 0.63 × lean mass − 4.1.

### Inputs and outputs for the liver GEM in the fasting state

During fasting conditions, the liver takes up gluconeogenic substrates and non‐esterified FAs and AAs and produces blood glucose (as an energy substrate for the brain), VLDL (as an energy substrate for the rest of the body), ketone bodies, and plasma proteins. The proteins secreted by the liver (mainly albumin) are not necessarily a net loss for the liver since protein can be recycled. However, in this study, the urea loss from urine was used as a proxy for the net loss of protein from the liver.

The input variables in our model are thus: (i) AAs, (ii) lactate, and (iii) FAs and glycerol. The output variables are as follows: (iv) glucose derived from gluconeogenesis and glycogenolysis and (v) ketone bodies as well as the measured VLDL secretion.

#### (i) AAs

In the fasting state, some AAs are released by muscle tissue. Pozefsky *et al* (1976) experimentally quantified the AA release from muscle tissue in the fasting state. They found that around 60% of all the AAs released from muscle are glutamine and alanine, which are the main substrates used for gluconeogenesis in the liver. These experimentally measured values were incorporated into the model based on the muscle mass of each subject.

Adipose tissue also releases AAs into the blood. Since the subjects in the present study had varying degrees of adiposity, it is important to know whether the release of AA differs between lean and obese subjects. Patterson *et al* (2002) found that although AA release is proportional to the amount of fat tissue a person has, it also depends on blood flow, which decreases as the amount of fat tissue increases. Thus, the release of AAs from adipose tissue is independent of obesity. Therefore, we included in the model an additional input of AAs based on adipose tissue mass. This contribution was calculated based on the study of Frayn and Karpe (Frayn & Karpe, 2014) where they measured how much blood flows in and out of adipose tissue (3–4 ml/min, 100 g fat tissue).

Another method (Ardilouze *et al*, 2004) provided information on a person's fat mass based on their BMI, gender, and age according to the formula: body fat percent = (1.2 × BMI) + (0.23 × age) − (10.8 × gender) − 5.4, where gender is 0 for female and 1 for male (Deurenberg *et al*, 1991). This resulted in an average body fat mass of around 15 kg which gave an average blood flow of the whole adipose tissue of around 31.5 l/h. Since Patterson *et al* (2002) provided the values for the release of AAs based on body fat (in μmol/l), the release of AAs by the adipose tissue (in mmol/h) was calculated for each subject and used as an input to the personalized models.

Muscle tissue and adipose tissue are not the only sources of AAs for the liver during starvation. It has been shown that rat liver catabolizes around 25% of all intracellular proteins during the first 24 h of starvation (Cuervo & Dice, 1996). In our present analysis, the total AAs released from muscle and adipose tissues do not seem to satisfy the liver demand for AAs. During 16 h of fasting, the urea excretion rate measured in humans was 392 ± 44 mmol urea/24 h (Norrelund *et al*, 2001). Assuming an average nitrogen content of 1.45 nitrogen atoms per AA and an average AA molar mass of 136.5 g/mol, the consumption of AAs in the liver after a 16‐h fast thus averaged close to 80 g/day (392 mmol/24 h × 136.5 g/mol/1,000/1.45 = 77.6 g AAs/day). This value was almost constant after 40 h of fasting (440 mmol/24 h), indicating maintained (or even increased) AA consumption in the fasting liver. The total amount of AAs released by muscle and adipose tissues was calculated to be close to 35 g/day indicating that the liver, in the present study, likely catabolizes itself in relatively large quantities—approximately 40–45 g/day. The AA composition of human liver has been measured by Benga and Ferdinand (Benga & Ferdinand, 1995). The molar ratios of the AAs in liver were incorporated into an additional input reaction to the model in order to achieve realistic AA net consumption values.

#### (ii) Lactate

Lactate is used as a gluconeogenic substrate in the liver. Wallace and Barritt (2005) claimed that the total amount of lactate produced by red blood cells, the kidney, the medulla, and the retina is around 40 g/day assuming resting conditions. In addition, an extra 40 g is produced by the rest of the body thus totaling around 80 g. This corresponds to around 3.3 g/h = 37 mmol/h and was used as an input to the model.

#### (iii) FAs and glycerol

Fatty acids are used by the liver for production of TG in VLDL. Glycerol is a by‐product of TG breakdown and subsequent FA release by adipose tissue and can be used as a gluconeogenic substrate. The FA and glycerol release from adipose tissue was estimated based on a study by McQuaid *et al* (2011), and values of FA and glycerol release from adipose tissue were retrieved for each subject in the fasting state. This average value was around 30 μmol/min/kg fat mass which is equal to 1.8 mmol/h/kg fat mass. Since the molar ratio of glycerol release to FA release is 1:3, the glycerol release was set as 0.6 mmol/h/kg fat mass. Both of these values were considered as upper bounds. However, Bickerton *et al* (2007) measured the total FA influx into muscle in fasting subjects and found that only around 4% of the FA released by adipose tissue was taken up by muscle. Thus, the released FA of 1.8 mmol/h was used as an input to the model.

#### (iv) Gluconeogenesis and glycogenolysis

Lactate, glutamine, alanine, and glycerol are the main gluconeogenic substrates. Another source of glucose is glycogen breakdown. McQuaid *et al* (2011) and Hellerstein *et al* (1997) reported that under normal overnight fasting conditions, the contribution of gluconeogenesis and glycogen breakdown to liver glucose output is roughly equal. McQuaid *et al* (2011) also found that glycogen breakdown is approximately 5.5 μmol/kg/min in humans after an overnight fast which corresponds to an average contribution from glycogenolysis of around 5.7 g glucose/h for the subjects in our study. The brain requires approximately 6 g glucose/h early in fasting when ketone body production is still low (Bourre, 2006). This suggests that during overnight fasting conditions, the total glucose output from the liver is in the order of 10–15 g/h and it is definitely higher than 6 g/h. In conclusion, an absolute minimum contribution of gluconeogenesis to glucose output was set as 16.7 mmol/h (3 g/h).

#### (v) Ketone bodies

The total ketone body production in obese humans increases dramatically up to around 60 mmol/h after 2–3 days of fasting and up to around 75 mmol/h after 17–24 days of fasting (Reichard *et al*, 1974). However during an overnight fast, the glycogenolysis should satisfy the majority of the brain's energy demand and the ketone body production rates for acetoacetate and beta‐hydroxybutyrate were therefore set at a lower bound of 0.1 mmol/h in the models.

### Personalized genome‐scale metabolic models for liver tissue

A functional GEM for hepatocytes in liver, *iHepatocytes2322,* was reconstructed based on hepatocyte‐specific proteomics data in Human Protein Atlas (HPA, http://www.proteinatlas.org) (Uhlen *et al*, 2015). Use of *iHepatocytes2322* in conjunction with flux balance analysis allowed for *in silico* metabolic simulation of liver for each subject involved in our study. We incorporated the measured/calculated uptake and secretion rate of the key metabolites into each GEM and predicted the intracellular liver fluxes of each patient. During the personalized simulation of liver tissue GEMs, we allowed the uptake of oxygen, phosphate, minerals, etc. (Dataset EV1) by the model and blocked the uptake of other metabolites since we simulated the fasting state. After setting all the bounds, we calculated the fluxes of all the subjects by minimizing the sum of fluxes, based on the assumption that the cells will reduce the pathway usage to a minimum for economic reasons (Dataset EV2). To test the robustness of the result, we also calculated the fluxes by random sampling without minimizing the flux sum and observed the same key results (Datasets E7, E8, E9 and E10).

To investigate the contribution of personalized inputs and outputs (uptake of FAs and VLDL secretion) to our conclusions, we performed a random control analysis (a random value with the range of the maximum and minimum value of all the patients). We found that when using random FA uptake or VLDL secretion alone as an input or output to our personalized models, the correlation between reactions carried by NNT and GSR and HS was significantly decreased (Datasets E11, E12, E13 and E14). Moreover, when both random FA uptake and VLDL secretion were used, the correlation became non‐significant (Datasets EV15 and EV16). Thus, we concluded that both personalized inputs and outputs are driving the conclusions reached in our study.

### Metabolomics data

Non‐targeted metabolite detection and quantification was conducted by the metabolomics provider Metabolon Inc. (Durham, USA) on fasting plasma samples collected from the subjects with varying degrees of HS. Samples were prepared using the automated MicroLab STAR^®^ system from Hamilton Company. A recovery standard was added before the first step in the extraction process for quality control purposes. To remove protein and dissociated small molecules bound to protein or trapped in the precipitated protein matrix, and to recover chemically diverse metabolites, proteins were precipitated with methanol under vigorous shaking for 2 min (Glen Mills GenoGrinder 2000) followed by centrifugation. The resulting extract was divided into four fractions: one for analysis by UPLC‐MS/MS with positive‐ion mode electrospray ionization, one for analysis by UPLC‐MS/MS with negative‐ion mode electrospray ionization, one for analysis by GC‐MS, and one sample reserved for backup.

Following log transformation, with the minimum observed value for each compound, Welch's two‐sample *t*‐test was used to identify the metabolites that differed significantly between subjects with high and low HS. During the identification of the significant metabolites as well as the significantly correlated metabolites, no data were imputed for the missing values. The correlation analysis between the metabolites was performed if both metabolites were detected in at least 30 subjects involving in our study.

### Mouse experiments

Twenty male C57BL/6J mice were fed a standard mouse chow diet (Purina 7012, Harlan Teklad) and housed in a 12‐h light–dark cycle. From the age of 8 weeks, mice were fed a Western diet (TD.88137, Harlan Laboratories, WI, USA) by separating into two groups. The mice were then divided into two groups of 10 mice. One group of mice was given the Western diet supplemented with NR (400 mg/kg/day) and serine (300 mg/kg/day) *la gavage* and NAC (1 g/l) in the drinking water for 14 days. The other group was only given the Western diet for the 14 days.

The mice were housed at the University of Gothenburg animal facility (Lab of Exp Biomed) and supervised by university veterinarians and professional staff. The health status of our mice is constantly monitored according to the rules established by the Federation of European Laboratory Animal Science Associations. The experiments were approved by the Gothenburg Ethical Committee on Animal Experiments.

### Lipid extraction and analysis

Lipids were extracted as described previously (Lofgren *et al*, 2012). Internal standards were added during the extraction. Lipids were analyzed using a combination of HPLC and mass spectrometry as described (Stahlman *et al*, 2013). Briefly, straight‐phase HPLC was used to purify ceramides (CER). Cholesteryl ester (CE), triacylglycerol (TAG), phosphatidylethanolamine (PE), phosphatidylcholine (PC), and sphingomyelin (SM) were quantified using a QTRAP 5500 mass spectrometer (Sciex, Concord, Canada) equipped with a robotic nanoflow ion source, TriVersa NanoMate (Advion BioSciences, Ithaca, NJ). CER were analyzed using reversed‐phase HPLC coupled to a triple‐quadrupole Quattro Premier mass spectrometer (Waters, Milford, MA, USA).

### Data availability

Supplemental information is provided in Datasets EV1, EV2, EV3, EV4, EV5, EV6, EV7, EV8, EV9, EV10, EV11, EV12, EV13, EV14, EV15 and EV16. The accession number for the raw and processed RNA‐seq data for liver tissue reported in this paper is available at Gene Expression Omnibus (GEO): GSE83322.

## Author contributions

AM developed the personalized liver models and analyzed the clinical data together with EB, CZ, and JN. JB coordinated the generation of the clinical data. JB, MA, PHRB, GFW, US, SS, BV, and M‐RT generated the VLDL kinetics and metabolomics data. MKi and MJS measured the expression of the genes in liver, and BMH and MU analyzed the data. MKl performed the mice supplementation study, and MS measured the hepatic lipid content. H‐UM performed the human serine supplementation study. NL, AH, and JL generated the MRS/MRI data. AM wrote the paper, and all authors were involved in editing the paper.

## Conflict of interest

AM, JB, and MU have filed a patent application about the use of the reported cocktail to treat metabolic diseases.

## Supporting information



Dataset EV1

Dataset EV2

Dataset EV3

Dataset EV4

Dataset EV5

Dataset EV6

Dataset EV7

Dataset EV8

Dataset EV9

Dataset EV10

Dataset EV11

Dataset EV12

Dataset EV13

Dataset EV14

Dataset EV15

Dataset EV16

Review Process File

Source Data for Figure 6
