# Supplementary material for: Personal model‐assisted identification of NAD+ and glutathione metabolism as intervention target in NAFLD
Source: Mol Syst Biol. 2017 Mar 2;13(3):916. doi: 10.15252/msb.20167422 (PMC5371732; doi:10.15252/msb.20167422)
Supplement: Supplementary file 17 — Review Process File [file MSB-13-916-s018.pdf]

Personal model-assisted identification of NAD+ and glutathione metabolism as intervention target in NAFLD

Adil Mardinoglu, Elias Bjornson, Cheng Zhang, Martina Klevstig, Sanni Söderlund, Marcus Ståhlman, Martin Adiels, Antti Hakkarainen, Nina Lundbom, Murat Kilicarslan, Björn M Hallström, Jesper Lundbom, Bruno Vergès, Peter Hugh R Barrett, Gerald F Watts, Mireille J. Serlie, Jens Nielsen, Mathias Uhlén, Ulf Smith, Hanns-Ulrich Marschall, Marja-Riitta Taskinen and Jan Boren

Corresponding author: Adil Mardinoglu, Chalmers University of Technology

Review timeline:

Submission date:	31 October 2016
Editorial Decision:	13 December 2016
Revision received:	29 December 2016
Accepted:	13 January 2017

Editor: Thomas Lemberger

Transaction Report:

Please note that the manuscript was previously reviewed at another journal and the reports were taken into account in the decision making process at Molecular Systems Biology. Since the original reviews are not subject to EMBO's transparent review process policy, the reports and author response cannot be published.

© European Molecular Biology Organization

EMBO PRESS

YOU MUST COMPLETE ALL CELLS WITH A PINK BACKGROUND ↓

PLEASE NOTE THAT THIS CHECKLIST WILL BE PUBLISHED ALONGSIDE YOUR PAPER

Corresponding Author Name: Adil Mardinoglu
Journal Submitted to: Molecular Systems Biology
Manuscript Number: MSB-16-7422

Reporting Checklist For Life Sciences Articles (Rev. July 2015)

This checklist is used to ensure good reporting standards and to improve the reproducibility of published results. These guidelines are consistent with the Principles and Guidelines for Reporting Preclinical Research issued by the NIH in 2014. Please follow the journal's authorship guidelines in preparing your manuscript.

A- Figures

1. Data

The data shown in figures should satisfy the following conditions:

- the data were obtained and processed according to the field's best practice and are presented to reflect the results of the experiments in an accurate and unbiased manner.
- figure panels include only data points, measurements or observations that can be compared to each other in a scientifically meaningful way.
- graphs include clearly labeled error bars for independent experiments and sample sizes. Unless justified, error bars should not be shown for technical replicates.
- if n< 5, the individual data points from each experiment should be plotted and any statistical test employed should be justified
- Source Data should be included to report the data underlying graphs. Please follow the guidelines set out in the author ship guidelines on Data Presentation.

2. Captions

Each figure caption should contain the following information, for each panel where they are relevant:

- a specification of the experimental system investigated (eg cell line, species name).
- the assay(s) and method(s) used to carry out the reported observations and measurements
- an explicit mention of the biological and chemical entity(ies) that are being measured.
- an explicit mention of the biological and chemical entity(ies) that are altered/varied/perturbed in a controlled manner.
- the exact sample size (n) for each experimental group/condition, given as a number, not a range;
- a description of the sample collection allowing the reader to understand whether the samples represent technical or biological replicates (including how many animals, litters, cultures, etc.).
- a statement of how many times the experiment shown was independently replicated in the laboratory.
- definitions of statistical methods and measures:
  - common tests, such as t-test (please specify whether paired vs. unpaired), simple χ2 tests, Wilcoxon and Mann-Whitney tests, can be unambiguously identified by name only, but more complex techniques should be described in the methods section;
  - are tests one-sided or two-sided?
  - are there adjustments for multiple comparisons?
  - exact statistical test results, e.g., P values = x but not P values < x;
  - definition of 'center values' as median or average;
  - definition of error bars as s.d. or s.e.m.

Any descriptions too long for the figure legend should be included in the methods section and/or with the source data.

Please ensure that the answers to the following questions are reported in the manuscript itself. We encourage you to include a specific subsection in the methods section for statistics, reagents, animal models and human subjects.

In the pink boxes below, provide the page number(s) of the manuscript draft or figure legend(s) where the information can be located. Every question should be answered. If the question is not relevant to your research, please write NA (non applicable).

USEFUL LINKS FOR COMPLETING THIS FORM

http://www.antibodypedia.com
http://1degreebio.org
http://www.equator-network.org/reporting-guidelines/improving-bioscience-research-repo
http://grants.nih.gov/grants/olaw/olaw.htm
http://www.mrc.ac.uk/Ourresearch/Ethicsresearchguidance/Useofanimals/index.htm
http://ClinicalTrials.gov
http://www.consort-statement.org
http://www.consort-statement.org/checklists/view/32-consort/66-title
http://www.equator-network.org/reporting-guidelines/reporting-recommendations-for-tun
http://datadryad.org
http://figshare.com
http://www.ncbi.nlm.nih.gov/gap
http://www.ebi.ac.uk/ega
http://biomodels.net/
http://biomodels.net/miriam/
http://jjj.biochem.sun.ac.za
http://oba.od.nih.gov/biosecurity/biosecurity_documents.html
http://www.selectagents.gov/

B- Statistics and general methods

Please fill out these boxes ↓ (Do not worry if you cannot see all your text once you press return)

1.a. How was the sample size chosen to ensure adequate power to detect a pre-specified effect size?	We designed proof of concept human study and included 6 subjects to the study. After seeing the significant effect, we decided to publish it in its current form.
1.b. For animal studies, include a statement about sample size estimate even if no statistical methods were used.	We included 10 animals in each group and have also verified the results in an independent experiment.
2. Describe inclusion/exclusion criteria if samples or animals were excluded from the analysis. Were the criteria pre-established?	No samples were excluded from the analysis.
3. Were any steps taken to minimize the effects of subjective bias when allocating animals/samples to treatment (e.g. randomization procedure)? If yes, please describe.	We have separeted animals in two two group and suplemented one group with the cocktail to see its effect on liver fat. The lipidomics were performed by a staff scientist with blinded samples.
For animal studies, include a statement about randomization even if no randomization was used.	We have separeted animals in two two group and suplemented one group with the cocktail to see its effect on liver fat. The person who handled the mice did not perform the lipidomics analyses of the samples.
4.a. Were any steps taken to minimize the effects of subjective bias during group allocation or/and when assessing results (e.g. blinding of the investigator)? If yes please describe.	The lipidomics analyses were performed with blinded samples. The clinical chemistry measurements were performed by a professional accredited clinical chemistry laboratory with blinded sampes. The measurement of liver fat were performed by specialized clinical staff without knowing anything about the ongoing clinical trial.
4.b. For animal studies, include a statement about blinding even if no blinding was done	We have not consider blinding of the investigator. The reason is that we wanted the same person to perform all la gavage treatments, in order to minmize variation.
5. For every figure, are statistical tests justified as appropriate?	Yes. We used appropriate statistical method.
Do the data meet the assumptions of the tests (e.g., normal distribution)? Describe any methods used to assess it.	Yes. The data meet the assumptions of the tests.
Is there an estimate of variation within each group of data?	Yes.
Is the variance similar between the groups that are being statistically compared?	Yes. We used appropriate statistical method.

C- Reagents

6. To show that antibodies were profiled for use in the system under study (assay and species), provide a citation, catalog number and/or clone number, supplementary information or reference to an antibody validation profile. e.g., Antibodypedia (see link list at top right), 1DegreeBio (see link list at top right).	Mouse diets are defined.
7. Identify the source of cell lines and report if they were recently authenticated (e.g., by STR profiling) and tested for mycoplasma contamination.	The health status of our mice is constantly monitored according rules established by the Federation of European Laboratory Animal Science Associations

* for all hyperlinks, please see the table at the top right of the document

D- Animal Models

8. Report species, strain, gender, age of animals and genetic modification status where applicable. Please detail housing and husbandry conditions and the source of animals.	The mice were housed at the Univ of Gothenburg animal facility (Lab of Exp Biomed) and supervised by university veterinarians and professional staff. The experiments were approved by the Gothenburg Ethical Committee on Animal Experiments. The mice were on C57BL/6J background from Janvier Labs, 8 weeks old when they were fed high-fat diet and 10 weeks when they started to receive la gavage feeding. Only males were used. The diets are defined in the manuscript.
9. For experiments involving live vertebrates, include a statement of compliance with ethical regulations and identify the committee(s) approving the experiments.	N/A
10. We recommend consulting the ARRIVE guidelines (see link list at top right) (PLoS Biol. 8(6), e1000412, 2010) to ensure that other relevant aspects of animal studies are adequately reported. See author guidelines, under 'Reporting Guidelines'. See also: NIH (see link list at top right) and MRC (see link list at top right) recommendations. Please confirm compliance.	The ARRIVE Guidelines (Animal Research: Reporting In Vivo Experiments) are followed. This detailed information is included in the application for the animal ethics. Thus, all experiments are well documented.

E- Human Subjects

11. Identify the committee(s) approving the study protocol.	The clinical study was approved by the Gothenburg Human Ethical Committee, and submitted to ClinicalTrials. Gov with the identifier NCT02599038.
12. Include a statement confirming that informed consent was obtained from all subjects and that the experiments conformed to the principles set out in the WMA Declaration of Helsinki and the Department of Health and Human Services Belmont Report.	Informed consents were obtained from all subjects and the experiments conformed to the principles set out in the WMA Declaration of Helsinki and the Department of Health and Human Services Belmont Report.
13. For publication of patient photos, include a statement confirming that consent to publish was obtained.	N/A
14. Report any restrictions on the availability (and/or on the use) of human data or samples.	Human samples can only be used in line with the approved ethical permission, and the data must be anonymous.
15. Report the clinical trial registration number (at ClinicalTrials.gov or equivalent), where applicable.	NCT02599038
16. For phase II and III randomized controlled trials, please refer to the CONSORT flow diagram (see link list at top right) and submit the CONSORT checklist (see link list at top right) with your submission. See author guidelines, under 'Reporting Guidelines'. Please confirm you have submitted this list.	N/A
17. For tumor marker prognostic studies, we recommend that you follow the REMARK reporting guidelines (see link list at top right). See author guidelines, under 'Reporting Guidelines'. Please confirm you have followed these guidelines.	N/A

F- Data Accessibility

18. Provide accession codes for deposited data. See author guidelines, under 'Data Deposition'.<br><br>Data deposition in a public repository is mandatory for:<br>a. Protein, DNA and RNA sequences<br>b. Macromolecular structures<br>c. Crystallographic data for small molecules<br>d. Functional genomics data<br>e. Proteomics and molecular interactions	The accession number for the raw and processed RNA-seq data for liver tissue reported in this paper is available at Gene Expression Omnibus (GEO): GSE83322.
19. Deposition is strongly recommended for any datasets that are central and integral to the study; please consider the journal's data policy. If no structured public repository exists for a given data type, we encourage the provision of datasets in the manuscript as a Supplementary Document (see author guidelines under 'Expanded View' or in unstructured repositories such as Dryad (see link list at top right) or Figshare (see link list at top right).	NA
20. Access to human clinical and genomic datasets should be provided with as few restrictions as possible while respecting ethical obligations to the patients and relevant medical and legal issues. If practically possible and compatible with the individual consent agreement used in the study, such data should be deposited in one of the major public access-controlled repositories such as dbGAP (see link list at top right) or EGA (see link list at top right).	NA
21. As far as possible, primary and referenced data should be formally cited in a Data Availability section. Please state whether you have included this section.<br><br>Examples:<br>**Primary Data**<br>Wetmore KM, Deutschbauer AM, Price MN, Arkin AP (2012). Comparison of gene expression and mutant fitness in Shewanella oneidensis MR-1. Gene Expression Omnibus GSE39462<br>**Referenced Data**<br>Huang J, Brown AF, Lei M (2012). Crystal structure of the TRBD domain of TERT and the CR4/5 of TR. Protein Data Bank 4O26<br>AP-MS analysis of human histone deacetylase interactions in CEM-T cells (2013). PRIDE PXD000208	NA
22. Computational models that are central and integral to a study should be shared without restrictions and provided in a machine-readable form. The relevant accession numbers or links should be provided. When possible, standardized format (SBML, CellML) should be used instead of scripts (e.g. MATLAB). Authors are strongly encouraged to follow the MIRIAM guidelines (see link list at top right) and deposit their model in a public database such as Biomodels (see link list at top right) or JWS Online (see link list at top right). If computer source code is provided with the paper, it should be deposited in a public repository or included in supplementary information.	The model used in this study has been deposited to Biomodels with the accession number MODEL1402200003.

G- Dual use research of concern

23. Could your study fall under dual use research restrictions? Please check biosecurity documents (see link list at top right) and list of select agents and toxins (APHIS/CDC) (see link list at top right). According to our biosecurity guidelines, provide a statement only if it could.	No
